# Supplementary material for: Mapping Cell-in-Cell Structures in Oral Squamous Cell Carcinoma
Source: Cells. 2023 Oct 8;12(19):2418. doi: 10.3390/cells12192418 (PMC10572403; doi:10.3390/cells12192418)
Supplement: Supplementary file 1 [file cells-12-02418-s001.zip › cells-2554835-supplementary.pdf]

SUPPLEMENTARY FILE

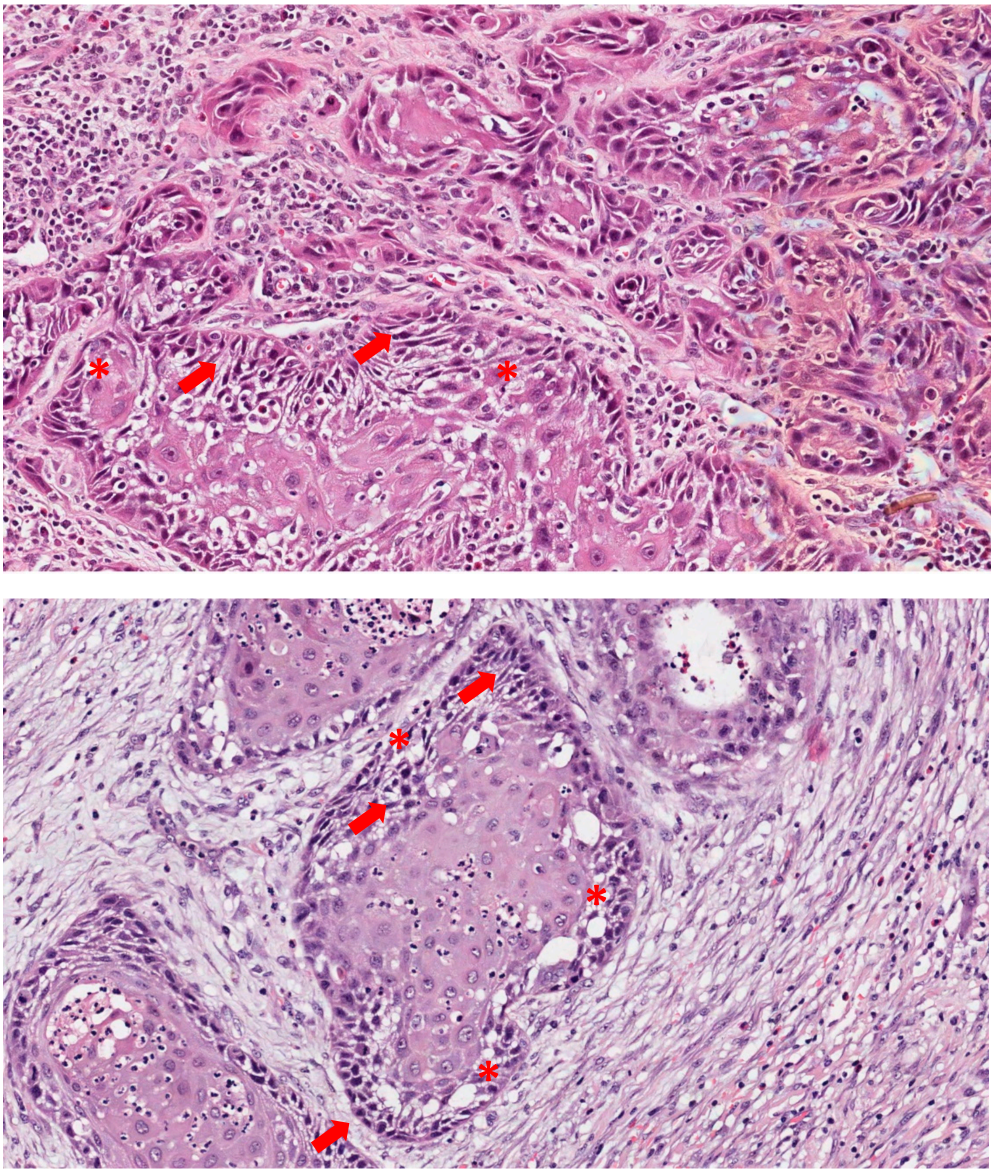

Supplementary Figure S1. Histomorphological aspects of oral squamous cell carcinoma. Islands of epithelial cells exhibiting polyhedral format with intercellular bridges. Especially in the periphery of the tumor islands, we also observe cells in a fusiform pattern. Cellular hyperchromatism (red arrows) and clear cytoplasm (*) may also be observed. Magnification: 400x.
